# Using Consensus Land Cover Data to Model Global Invasive Tree Species Distributions

**DOI:** 10.3390/plants11070981

**Published:** 2022-04-04

**Authors:** Fei-Xue Zhang, Chun-Jing Wang, Ji-Zhong Wan

**Affiliations:** 1 State Key Laboratory of Plateau Ecology and Agriculture, Qinghai University, Xining 810016, China; zfx660109@163.com; 2 College of Agriculture and Animal Husbandry, Qinghai University, Xining 810016, China; 2018990003@qhu.edu.cn

**Keywords:** consensus land cover data, global invasive tree species, Maxent, AUC, species distribution models

## Abstract

Invasive tree species threaten ecosystems, natural resources, and managed land worldwide. Land cover has been widely used as an environmental variable for predicting global invasive tree species distributions. Recent studies have shown that consensus land cover data can be an effective tool for species distribution modelling. In this paper, consensus land cover data were used as prediction variables to predict the distribution of the 11 most aggressive invasive tree species globally. We found that consensus land cover data could indeed contribute to modelling the distribution of invasive tree species. According to the contribution rate of land cover to the distribution of invasive tree species, we inferred that the cover classes of open water and evergreen broadleaf trees have strong explanatory power regarding the distribution of invasive tree species. Under consensus land cover changes, invasive tree species were mainly distributed near equatorial, tropical, and subtropical areas. In order to limit the damage caused by invasive tree species to global biodiversity, human life, safety, and the economy, strong measures must be implemented to prevent the further expansion of invasive tree species. We suggest the use of consensus land cover data to model global invasive tree species distributions, as this approach has strong potential to enhance the performance of species distribution modelling. Our study provides new insights into the risk assessment and management of invasive tree species globally.

## 1. Introduction

As biodiversity has changed worldwide, progressively more non-native invasive trees now occupy the ranges and habitats of local plant species to varying degrees and are destroying the long-term ecological balance [1]. Invasive species, i.e., non-native species intentionally or unintentionally introduced from outside the local scope, can destroy the local ecological balance and pose a serious threat to the economy and ecology by crowding out the native plants in the environment [1,2,3].

Owing to invasive trees having strong stress tolerance and responding quickly to changes in the external environment, they have increasingly become one of the most urgent problems worldwide in the 21st century [4]. In addition, invasive species usually hinder local species from obtaining their required nutrients, water, and light while changing the soil chemistry, hydrological patterns, and moisture-holding capacity of the invaded area, thus, changing the dynamics of plant communities [2,3]. Although the abundance and frequency of most non-native species are very low, some species can become invasive species through their rapid spread. Some species can even change the local plant species compositions, inhibit local tree regeneration, and alter ecosystem functions. 

For example, *Hiptage benghalensis* is a weed in the tropical rain forests of Australia and is highly invasive to Mauritius and Réunion, where it thrives in the dry lowland forests forming impenetrable stands of shrubs and suffocating the local vegetation [5,6,7]. Other studies have shown that some species may invade only a limited area but are likely to expand and cause further substantial damage [6].

Prediction of the potential distribution of non-native invasive species can help to prioritize the identification of invasive species and sites for early detection and indicate the best preventive measures [1]. Therefore, there has been an urgent need to develop reliable intrusion prediction methods to adjust monitoring and/or management policies and reduce the risk of invasive tree species spread and range expansion [2,8]. The relationship between plant traits and the environment has been linked to non-native invasive species, as rapid urbanization can homogenize biodiversity, reduce habitat heterogeneity, and promote the invasion of non-native species [9]. 

However, more research on invasive species has focused on predicting the distribution of non-native invasive species through the use of climate change, human activities, soil, and, more recently, land use as environmental variables in non-native invasive species distribution models [10]. Although the effects of various abiotic and biological factors on invasive trees have been well studied, little is known about the effects of consensus land cover data on plant invasions [11]. 

Recent research has shown that consensus land cover data can improve spatial resolutions and limit the geographical deviation of spatial biodiversity knowledge, which is one of the key data and modelling challenges in biogeography, ecology, conservation, and related fields; however, it has remained unknown whether it can accurately predict the distribution of invasive species [12]. At the same time, the importance of consensus land cover to the richness of local alien invasive trees is unclear.

Land cover plays an important role in Earth’s systems. It affects the flows of energy and material [7] and forms an important interface between human activities and natural environments [13]. Consensus land cover is a product derived from multiple global remote sensing, which integrates four global land-cover products. In addition, it offers accuracy-weighted consensus information on the prevalence of 12 land cover classes within every nominal 1 km pixel across the globe. Compared with the four base products, it better captures the land-cover information contained in the fine-grain validation data for all classes combined and for most individual categories. It also has the highest sensitivity and overall accuracy for detecting the presence of each fine-grain land-cover class. 

Consequently, it surpasses the single basic product in the ability to capture subpixel land cover information and the practicability of modelling species distributions [12]. Land cover information helps to improve the spatial resolution and limit the geographical deviation of spatial biodiversity knowledge, which is one of the key data and modeling challenges in biogeography, ecology, conservation, and beyond [14]. For example, land cover data have been incorporated into an inductive model (species distribution model), which correlates the presence/absence or abundance of species in different sites with the environmental conditions of those sites [15] to improve the accuracy of the model [16].

Owing to the differences in physiological characteristics between different organisms, different consensus land cover types are associated with great differences in plant invasion [10,17]. However, it is unclear whether this model is applicable to the species richness of local non-native plants [18]. For example, some invasive species that are suitable to grow in grasslands, wetlands, and tundra have low frequencies in coniferous forests [17]. Consensus land cover data has been demonstrated to have the highest sensitivity and overall accuracy in detecting each fine-grained land cover classes. For example, the deductive and inductive model established by Mao et al. with consensus land cover data had the highest or second highest accuracy in simulating bird species distributions [12]. 

Consensus land cover data have also been analyzed with a new model for assessing the current situation and trends in biodiversity, ecosystems, and climates [12]. Consensus land cover has been shown to be indispensable in assessing the earth’s ecosystem, as it reflects the flow and transmission of energy and material in the ecosystem. Moreover, consensus land cover has also been crucial for the sustainable development of biodiversity and effective resource allocation management [12,19]. 

In both aquatic and terrestrial environments, invasive species are causing global problems, and there is an urgent need to better predict their invasiveness [20]. To reduce invasions by non-native invasive trees, countries must recognize that non-native species pose a threat to their environment and economy and then adopt strong border control policies and establish improved systems of non-native species management laws and regulations, so as to improve the prevention and awareness of different groups of non-native invasive species, thus, protecting global ecological security [21,22].

We selected 11 representative invasive tree species with serious documented invasions into global environments from a list of 100 of the world’s worst invasive non-native specialties as assessed by a global expert group [23]. Compared with invasive herbs and shrubs, invasive tree species pose the greatest ecological, economic, and human survival impacts, because invasive tree species have a higher tolerance to habitats relative to shrubs and herbs, and they can therefore survive even in very unstable environments. In addition, invasive trees can occupy a large area of the growth area traditionally occupied by local species, thus, blocking the light and absorbing high levels of nutrients and water, thereby contributing to the rapid extinction of local species and exacerbating the difficulty of controlling these invasive tree species [24,25]. 

The present study used consensus land cover data as the prediction variable and the Maxent maximum entropy model to model the distribution of these 11 invasive tree species and evaluate whether this information can be used to accurately predict the distribution of the 11 tree species across 12 consensus land-cover classes. Specifically, the present work accomplished the following aims: (a) the area under the curve (AUC) and omission rates of 11 invasive tree species were used to test whether consensus land cover data can predict the potential distribution of invasive species; (b) the most important driving factors and effective indicators for the distribution of invasive tree species in the consensus land cover data were explored; and (c) the responses of invasive tree species distributions to land cover changes were determined.

## 2. Materials and Methods

### 2.1. Occurrence Data

We compiled a list of 100 of the world’s most invasive non-native specialties from the Invasive Species Specialist Group (ISSG) of the Global Invasive Species Database (GISD). We determine the 11 most representative non-native invasive tree species [10,23], they are *Ligustrum robustum*, *Cinchona pubescens*, *Morella faya*, *Miconia calvescens*, *Cecropia peltate*, *Spathodea campanulate*, *Melaleuca quinquenervia*, *Schinus terebinthifolia*, *Acacia mearnsii*, *Leucaena leucocephala*, and *Pinus pinaster*. The distribution data of these 11 invasive tree species were compiled from four online databases, respectively: (a) the Global Biodiversity Information Facility (GBIF; https://www.gbif.org (accessed on 23 December 2021)), (b) the IUCN/SSC ISSG [10,23], (c) LIFEMAPPER (https://lifemapper.ku.edu (accessed on 23 December 2021)), and (d) SPECIESLINK (www.splink.cria.org.br (accessed on 23 December 2021)). All extracted data were resampled with a resolution of 2.5 arc-minutes (about 5 km at the equator). The downloaded data were sorted and checked, and duplicate records were deleted in order to reduce sampling error and ensure the accuracy of predictions based on the model. Ultimately, we obtained 390,000 data points and were able to run Maxent.

### 2.2. Land Cover Data

Data describing consensus land cover were obtained from EarthEnv (https://www.earthenv.org/landcover (accessed on 26 December 2021)), which provides two versions of consensus land cover data. We selected the complete version of land cover data, and the full version is the dataset integrating GlobCover (2005-06; v2.2), the MODIS land-cover product (MCD12Q1; v051), GLC2000 (global product; v1.1), and DISCover (GLCC; v2). 

Each dataset contains 12 data layers, each of which provides consensus information on the prevalence of one land-cover class. All data layers contain unsigned 8-bit values, and the valid values range from 0 to 100, representing the consensus prevalence in percent. All data layers had a spatial extent from 90° N to 56° S and from 180° W to 180° E and a spatial resolution of 30 arc-seconds per pixel (approximately 1 km per pixel at the equator) [12]. The 12-consensus land-cover classes were evergreen/deciduous needleleaf trees, evergreen broadleaf trees, deciduous broadleaf trees, mixed/other trees, shrubs, herbaceous vegetation, cultivated and managed vegetation, regularly flooded vegetation, urban/built-up, snow or ice, barren, and open water. 

Previous studies have shown that by comparing the consensus land cover data with four basic land cover category products, the consensus land cover data set successfully retains more accurate land cover information from individual basic data products and reduces the inaccuracy of land cover information [12]. Thus, land cover data can be reasonably postulated to accurately predict the distribution of invasive tree species, with consensus land cover data serving as important information to predict the distribution of invasive tree species. 

Consensus land cover data have been shown to possibly have a significant impact on regional and seasonal scales, thus, further affecting the distribution of invasive tree species, and for land cover data in the original ecological geographical form, each land cover category is closely related to the management mode of ecosystems [7,12,26]. First, we need to convert the TIF files of the 12 downloaded consensus land cover data into BIL files in GIS software. Secondly, we need to convert the 12 BIL files into ASC files in DIVA-GIS software (http://www.diva-gis.org/download (accessed on 17 May 2021)), because in Maxent model, only CSV files of species distribution data and ASC files of environmental variables can be put in. Finally, we used the distribution latitude of 11 invasive tree species as the response variable and 12 consensus land cover classes as the prediction variable for running the Maxent model.

### 2.3. Modelling Approach and Evaluation

The maximum entropy model Maxent is generally considered as a model with high performance in predicting the distribution of invasive trees [27]. Here, we established a logistic regression model with 11 invasive tree species distribution data as response variables and 12 consensus land-cover classes as prediction variables. The data for the invasive tree species distribution was divided into a random training test set (auctest, 75%) and a test model set (auctrain, 25%). We set the regularization multiplier to 2 and the number of replicates to four. 

To limit other sources of variability in the analysis, the default values of other parameters were used. We used the area under the receiver operating characteristic (ROC) to evaluate the accuracy of model performance [27,28,29,30]. AUC values were used to evaluate the accuracy of the model, with values ranging from 0 to 1, and the larger the value, the more the species distribution deviated from a random distribution (AUC = 0.5). The greater the correlation between variables and the model, the higher the accuracy of the model. In this study, the model performance evaluated according to AUC values was divided into five categories: failure (0.5–0.6), average (0.6–0.7), good (0.7–0.8), very good (0.8–0.9), and outstanding (0.9–1) [2]. 

However, when the AUC value was independent of the size of the data set, the index can be inaccurate, because it may ignore the predicted probability value, goodness of fit, and the spatial range of the model. We learned that using predefined criteria (such as AUC scores) to evaluate model performance may not always be optimal, because many alternative species distribution models may produce similar results [31,32], and the results may be particularly chaotic if the training data are unbalanced [33]. Therefore, as a further performance test, we analyzed the omission rates, which may be a better way to understand the reliability of model results [34,35]. The lower the omission rate, the higher the prediction accuracy of Maxent [29]; therefore, the use of this approach in combination with AUC enables better evaluations of the accuracy of model performance.

### 2.4. Effects of Environment on Global Invasive Tree Species Distributions

We evaluated the impact of 12 consensus land-cover classes on the distribution of invasive tree species. First, we determined the dominant factors shaping the distribution patterns of 11 invasive tree species based on the size of the contributions rates of each land-cover class for the 11 invasive tree species distributions. Second, we used the response curve to check the response of invasive tree species distributions to the consensus land-cover classes to thus judge the suitable distribution area of invasive tree species. This information can be used to protect the potential distribution area of invasive tree species in order to prevent invasion by invasive tree species. In this way, we can adopt more powerful management measures to prevent the further expansion of invasive tree species and limit their serious negative consequences.

## 3. Results

### 3.1. Importance of Validation Variables in Invasive Species Distribution Model

In the final Maxent model, we analyzed 390,000 distribution datapoints and 12 consensus land-cover classes as model parameters. Based on the ROC curve generated from the test data obtained by segmenting the training data, we determined that most of the model tables of these 10 non-native invasive tree species perform well (Table 1). The AUCs of 10 of the species were as follows: *C. pubescens*, 0.968; *M. faya*, 0.957; *M. calvescens*, 0.954; *C. peltata*, 0.950; *L. robustum*, 0.975; *S. campanulata*, 0.912; *M. quinquenervia*, 0.901; *S. terebinthifolia*, 0.843; *A. mearnsii*, 0.803; *L. leucocephala*, 0.786. 

However, the performance the model for *P. pinaster* was poor, with an AUC value of only 0.598 (Table 1). At the same time, we observed that the omission rate of *P. pinaster* was 0.446, which was too high, demonstrating that the validation of this model was not acceptable in *Pinus pinaster* (Table 1). In general, the model was reliable in predicting the habitat suitability of invasive tree species distributions, and the output of the model was close to the real probability distribution. Similarly, the omission rate showed that the observation results of the model were consistent with the prediction results and also supported the accuracy of the model when only the existing data were used to predict the suitable habitats. However, the performance of the model for *P. pinaster* was poor and merits specific follow up analysis.

We found that the average contribution of land cover class to model performance differed among the 11 invasive trees (Table 2). The Open Water cover class contributed the most to the distributions of seven species (32.62–74.57%), namely *C. peltata*, *L. robustum*, *M.quinquenervia*, *A. mearnsii*, *S. campanulata*, *S. terebinthifolia*, and *L. leucocephala*. 

Among these species, Evergreen Broadleaf Trees land cover contributed the second most to four species distributions (33.52–75.35%), including *M. quinquenervia*, *C. peltata*, *M. calvescens*, and *C. pubescens* (Table 2). Thus, Open Water and Evergreen Broadleaf Trees land cover classes had a greater impact on the distribution of invasive trees, and other effects of land cover classes were relatively small, which also showed that the land cover classes of Open Water and Evergreen Broadleaf Trees were the most effective indicators of the distribution of these invasive tree species.

### 3.2. Responses of Invasive Plant Species Distributions to Land Cover Changes

**Figure 1:**
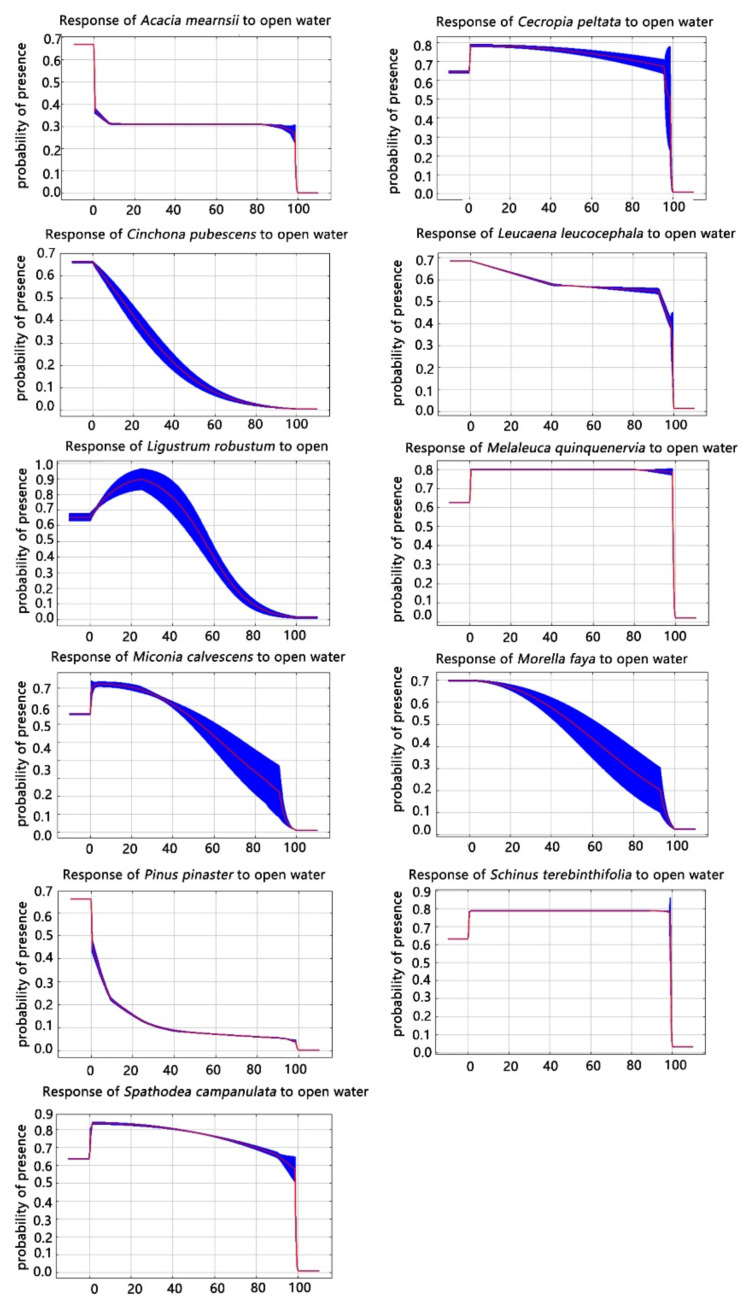
Response curves of 11 invasive tree species distributions to the open water land cover class.

According to their contribution rates, Open Water and Evergreen Broadleaf Trees cover classes were the most powerful predictors of the distribution of non-native invasive tree species. We found that the distribution of these 11 invasive trees had different responses to Open Water (Figure 1). When the probability of the existence of an invasive species was greater than 0.5, the corresponding prediction variables were consistent with a suitable habitat for invasive tree species, with valid values ranging from 0 to 100, representing the consensus prevalence in percent. 

**Figure 2:**
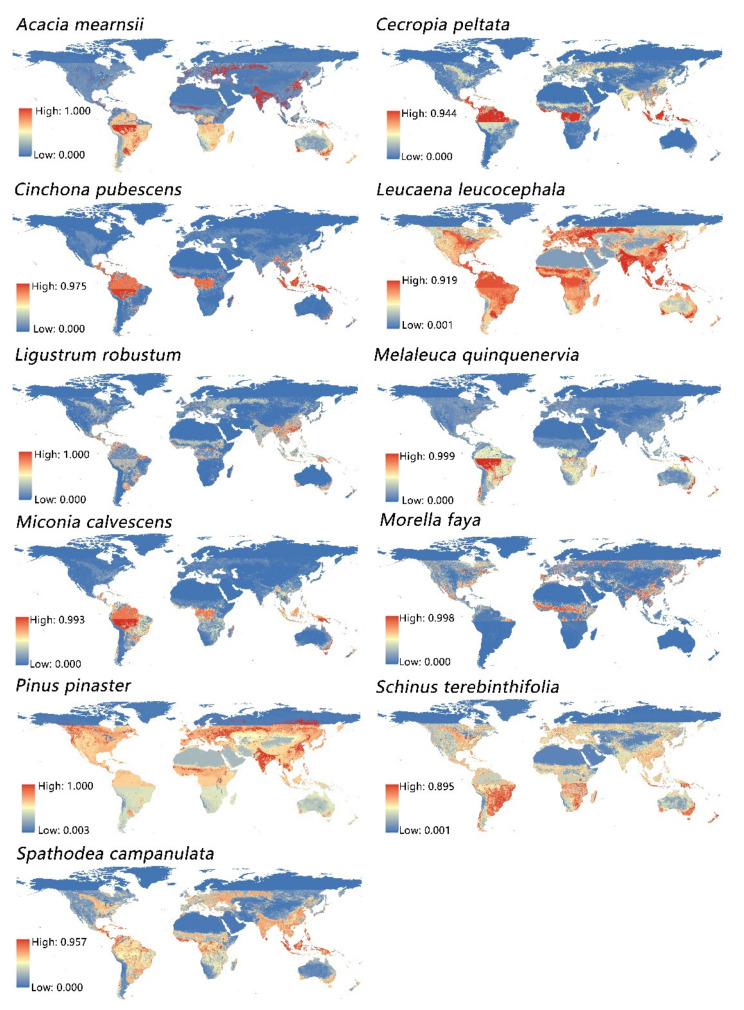
Hot spots of the probability distribution of 11 invasive tree species inferred to be influenced by the consensus land cover data. Red represents the high distribution probability of invasive tree species, and blue represents the low distribution probability of invasive tree species. The probability of distribution from blue to red increases gradually.

As shown in Figure 2, for *C. peltata*, *S. campanulata*, *M. quinquenervia*, *S. terebinthifolia*, and *L. leucocephala* with the increase of open water prevalence, the distribution probability of these five species was high. Thus, these five species were greatly affected by open water, and these five species were therefore predicted to be suited to inhabiting areas near open water. In addition, the distribution probabilities of *C. pubescens*, *M. calvescens* and *M. faya* gradually decreased with the increased prevalence of open water, which indicates that these four invasive trees were negatively affected by open water and not suited to survive near open water. 

However, the average contribution rate of the Open Water class to the distribution of *P. pinaster* was 53.40%, while the response curve shows that the species was less affected by the Open Water class, which reflects that the validation of the land cover class model for *P. pinaster* was inadequate. When the prevalence of open water was 15–40, the distribution probability of *L. robustum* was the highest. When the open water prevalence was 0–100, the distribution probability of *A. mearnsii* was always 0.3, which was low, indicating that the distribution probability of *A. mearnsii* responds little to the prevalence of open water (Figure 1).

### 3.3. Potential Distribution of Invasive Tree Species

Comparison of the distribution maps of 11 invasive tree species revealed that most invasive species were distributed near the equator, mainly in Central South America, Southeast Asia, eastern Australia, Central Africa, and Southeast North America (Figure 2). Specifically, *A. mearnsii* was mainly distributed in South America, Africa, India, Southeast China, and central Europe, while *C. peltata*, *C. pubescens*, and *M. calvescens* were mainly distributed near the equator, concentrated in northern South America, Central Africa, and Southeast Asia. The distribution probability of *L. leucocephala* was very wide across the probability map, covering almost the entire world, mainly in South America, Central Africa, Southeast Asia, India, eastern China, western Russia, Europe, and the central part of the United States. 

*L. robustum* was mainly distributed in southern China and northwestern Southeast Asia. *M. quinquenervia* was mainly distributed in the middle of South America and southeastern Australia, with lower probabilities in other regions. *M. faya* was mainly distributed in southeastern China and the southeastern United States. *S. terebinthifolia* was mainly distributed in eastern South America, southern Africa, and southeastern Australia. *S. campanulata* was mainly distributed in South America, Africa, India, Southeast China, and northern Russia. It was not difficult to determine that the climate and terrain of these 11 invasive trees are complex and diverse; however, the tropical and subtropical species present particularly high-risk areas for the introduction and establishment of these invasive trees.

## 4. Discussion

Although Maxent has been widely used in the modelling of plant invasions, the value of using Maxent with land cover data has not been studied. Our study used Maxent modelling to investigate the response of the distribution of invasive tree species to consensus land cover data [36]. The suitable habitat of invasive species predicted by the model was highly consistent with the existing records. Among the 11 invasive tree species investigated, the average AUC of seven species was more than 0.9, indicating the excellent predictive value of the model for these species. Two of the invasive species had AUC values of 0.75–0.85, indicating good model performance. However, the AUC value of *P. pinaster* was 0.598, indicating that the model cannot accurately predict the potential distribution of this species (Table 1). 

*P. pinaster* is native to the Mediterranean basin and had been planted in temperate areas inside and outside of its natural range for many reasons [36]. It can easily reproduce almost anywhere it is planted. In many places, it invades the natural areas of shrubs, forests, and grasslands and thus destroys local habitats [37]. *P. pinaster* has been shown to form dense stands of shrubs, block local plants from obtaining light and nutrients, change local fire and hydrological characteristics, and alter the habitat of many animal species [36,37]. We suspect that because *P. pinaster* has strong regenerative ability and fast propagation and invasion speed, the model may not be able to capture a suitable distribution area for this species. 

There are several potential explanations for why the Maxent model did not provide a strong validation with *P. pinaster* [32]: (1) With the expansion of population ranges, owing to imbalances with environmental conditions, the current distributions may not reflect the realization of niches [12]. (2) There are inconsistencies in the class definitions among evaluated products and the validation data (i.e., NLCD2006), and there is a lower prevalence or smaller patch sizes of these classes [12]. (3) Land cover classification schemes generally do not consider the habitat associations of specific species [12]. 

Therefore, land cover classifications may not correspond well to the preferred habitat types of species, and the combination of species and land cover classifications may not perfectly represent the habitat needs of species. Thus, there is a reasonable explanation for the results of the present study. Previous research has shown that the number of occurrence records can have a strongly negative impact on Maxent’s simulated distribution of invasive species [38]. In this paper, the occurrence records of *P. pinaster* reached 250,000. The uncertainty of sampling may reasonably increase as the number of occurrence records increases, indicating that the model cannot predict the suitable and unsuitable habitats of *P. pinaster*.

In this study, Open Water and Evergreen Broadleaf Trees were the dominant classes containing the distribution of invasive species, and other land cover classes had little potential impact on the distribution of invasive tree species. As is widely known, water changes during plant growth are among the most important monitoring parameters in plant research [39]. Open water land cover indicates that such areas provide suitable temperatures and water for organisms inhabiting the surrounding environment within a certain space and time range [38,40,41].

Some studies have also shown that the temperature and temperature difference around open water are much more stable than those around baren and urbanized land [42]. Therefore, the model shows that open water contributes considerably to the number of invasive species. In such areas, open water also provides a suitable habitat for a number of invasive species, thus, changing the distribution and richness of native plants and increasing competition against native plants. Invasive trees not only have a great demand for water but also absorb, store, and return water to the atmosphere—a particularly important series of ecological processes. In addition, open water may have some indirect impact on the response of vegetation and climate. As the distance from the open water increases, the response of vegetation and climate gradually decreases, thus, affecting the distribution of invasive tree species.

We found that the Open Water land cover class had the strongest explanatory power for the distributions of *L. robustum*, *S. campanulate*, *M. quinquenervia*, *S. terebinthifolia*, *A. mearnsii*, and *L. leucocephala* (Figure 2). *S. campanulata* is an evergreen tree native to West Africa that prefers humid habitats and grows best in sheltered tropical areas [43]. *L. robustum* is a highly invasive small tree inhabiting areas near the Indian Ocean that strongly prefers humid and dark environment [44]. 

Thus, on the marine islands it has invaded, this species destroys the regeneration of primary forests and threatens the local plant diversity. *M. quinquenervia* tends to invade wetlands, and through its rapid invasion, it has changed the ecosystems of swamps [45]. *S. terebinthifolia* is an aggressive evergreen shrub or small tree, 3–7 m high, that grows in various soil types and prefers local sunlight but generally grows in cool habitats [46]. Thus, the invasive tree species we investigated appeared to be enriched for having high water requirements, which has been noted for tree species with a serious tendency towards invasion.

Through our research, we also found that evergreen broadleaf trees also had a great impact on invasive tree species distributions. Our research showed that evergreen broadleaf trees play a key role in predicting the distributions of *C. pubescens*, *M. calvescens*, and *C. peltata*. *M. quinquenervia*. *C. pubescens*, *M. calvescens*, and *C. peltata* are all widely planted tropical forest tree species. They prefer habitats with sufficient rain and heat, invade a variety of forest and non-forest habitats, and have low requirements for the environment otherwise. We concluded that the land cover classes of Open Water and Evergreen Broadleaf Trees may be key to the spread of these invasive tree plants.

However, in this study, we found that other consensus land cover classes contributed little to the model. For example, Mixed/Other Tree, Barren, Shrubs, Herbaceous Vegetation, and Snow/Ice each exhibited very low contribution rates, as expected. In this paper, we examined the distributions of invasive tree species. Trees themselves have high environmental requirements. They need sufficiently large sites and sufficient water to achieve rapid growth. For example, consensus land-cover classes, such as glaciers and barren land were poor environments, with low biodiversity, small populations, low levels of urbanizations, and an inaccessibility to invasive tree species, thus, preventing their invasion.

The present research shows that invasive trees were more likely to be introduced, become established, and spread in tropical or subtropical areas [5]. Most of these 11 invasive tree species are native to tropical and subtropical areas of South America, southern Europe, East Asia, and South Asia. Previous research has also shown that some species had low habitat requirements, possibly due to their own physiological characteristics and strong adaptability to the surrounding environment and their ability to grow and spread rapidly in unfamiliar environments [4]. 

Most of these 11 invasive trees were concentrated in tropical or subtropical areas, because they all prefer areas with the same periods of rain and heat, more precipitation, and high temperatures. *L. leucocephala* and *S. terebinthifolia* are widely distributed and present almost worldwide. Thus, the morphological and physiological advantages of these three invasive tree species, such as their short life cycle, strong fecundity, strong transmission ability, and strong phenotypic plasticity, may enable them to survive under adverse climatic conditions. 

For example, *L. leucocephala* was initially introduced by various countries as a beneficial tree species; however, gradually the environmental damage caused by invasive species appeared. This spineless tree species can form dense single species stands of shrubs. Once established, it is difficult to eradicate invasive tree species that threaten local plants. These 11 invasive trees are widely distributed. In many places, they invade natural areas occupied by shrubs, forests, and grasslands, imposing great harm on the security of ecosystems and human society. In addition, 12 land cover classes were used as prediction variables to simulate the distribution of invasive species. 

Thus, we found that Open Water, Evergreen Broadleaf Trees, Urban or Built-up, and Regularly Flooded Vegetation cover classes were the areas in which invasive species tended to be distributed. Therefore, we need to vigorously protect the predicted potential distribution areas based on this consensus land-cover class data and take timely control and preventive measures. Otherwise, the economic loss and negative impact of invasive weeds on food security, biodiversity, and ecosystem services may increase dramatically in the near future.

Although this study used consensus land cover data to predict the distribution of invasive species, the Maxent model still had limitations. First, a limitation of the consensus dataset was its low temporal resolution, which may limit some critical applications in biodiversity or analyses of ecosystem change in the short term [47]. Second, the sample size of 11 invasive trees species is too small to make broad conclusions, and the distribution of data of each species was uneven. Some species reached 250,000 data points, while other species had only a few hundred distribution data points, which is likely to affect the accuracy of the model predictions [32].

Finally, this study only considered consensus land cover classes in the model analyzed and did not consider other habitat determinants, such as topography, soil characteristics, diffusion capacity, biological interactions (such as promotion and competition), and vector-driven species invasion. This study was conducted as part of an ongoing long-term study [10], and thus we will consider the use of other variables, including topographic and soil characteristics, diffusion capacity, and biological interactions, to obtain more accurate predictions in the near future.

## 5. Conclusions

Consensus land cover data were used to model global invasive tree species distributions, revealing that Maxent was accurate in predicting the distribution of invasive tree species. In addition, we also found that the land cover classes of Open Water and Evergreen Broadleaf Trees had strong relationships with the predicted species distributions.

According to the predictions of the potential distributions of these 11 invasive tree species and their response to consensus land cover classes, invasive tree species were more inclined to tropical and subtropical areas because tropical and subtropical areas had sufficient rain and heat and many forest resources, which also provide sites for invasive species. Accordingly, policy makers must strengthen management in areas vulnerable to invasion and formulate effective strategies to prevent the further expansion of invasive species.

## Figures and Tables

**Table 1:** Area under the curve (AUC) and omission rate values for 11 invasive tree species.

Species	Number of Occurrences	Omission Rate	AUC
*Ligustrum robustum*	109	0.065	0.975
*Cinchona pubescens*	627	0.065	0.968
*Morella faya*	799	0.078	0.957
*Miconia calvescens*	93,298	0.076	0.954
*Cecropia peltata*	2994	0.097	0.950
*Spathodea campanulata*	3039	0.149	0.912
*Melaleuca quinquenervia*	5732	0.167	0.901
*Schinus terebinthifolia*	7194	0.227	0.843
*Acacia mearnsii*	20,529	0.301	0.803
*Leucaena leucocephala*	12,747	0.299	0.786
*Pinus pinaster*	25,1158	0.446	0.598

**Table 2:** Contribution of consensus land-cover classes to the distributions of 11 invasive tree species. B, Barren; CAMV, Cultivated and Managed Vegetation; DBT, Deciduous Broadleaf Trees; EBT, Evergreen Broadleaf Trees; EDNT, Evergreen/Deciduous Needleleaf Trees; HV, Herbaceous Vegetation; MOT, Mixed/Other Trees; RFV, Regularly Flooded Vegetation; S, Shrubs; SI, Snow/Ice; UBU, Urban/Built-up; and OW, Open Water.

Species	B	CAMV	DBT	EBT	EDNT	HV	MOT	RFV	S	SI	UBU	OW
*Morella faya*	1.259	1.121	30.605	1.832	1.505	0.774	19.344	11.955	3.864	0.001	10.998	16.742
*Melaleuca quinquenervia*	1.240	0.304	0.290	33.523	0.238	0.075	0.293	2.185	0.657	0.000	25.392	35.804
*Ligustrum robustum*	5.426	21.347	1.457	9.089	1.339	2.209	5.173	6.390	3.091	0.016	11.040	33.423
*Cinchona pubescens*	0.996	0.802	1.937	75.353	0.454	0.036	0.915	3.082	0.101	0.009	0.427	15.888
*Cecropia peltata*	2.589	3.760	0.857	45.535	1.055	0.449	0.779	6.619	1.331	0.067	4.340	32.619
*Leucaena leucocephala*	4.736	3.577	0.300	0.086	0.915	0.629	0.610	13.905	0.348	0.004	0.323	74.568
*Pinus pinaster*	1.033	1.404	0.057	0.026	31.566	0.084	0.419	9.719	0.250	0.000	2.039	53.401
*Acacia mearnsii*	0.302	16.582	0.060	17.981	0.009	0.006	0.176	0.946	0.130	0.000	22.344	41.463
*Miconia calvescens*	1.775	1.075	0.510	68.729	0.603	0.581	0.666	4.090	0.304	0.014	1.641	20.012
*Schinus terebinthifolia*	5.121	0.338	0.118	0.491	0.619	0.967	1.723	11.807	1.022	0.003	10.408	67.385
*Spathodea campanulata*	7.228	3.382	0.413	5.157	1.158	2.202	2.726	11.884	1.494	0.060	2.809	61.488

## Data Availability

Not applicable.
